# Lessons learned about [F-18]-AV-1451 off-target binding from an autopsy-confirmed Parkinson’s case

**DOI:** 10.1186/s40478-017-0482-0

**Published:** 2017-10-19

**Authors:** Marta Marquié, Eline E. Verwer, Avery C. Meltzer, Sally Ji Who Kim, Cinthya Agüero, Jose Gonzalez, Sara J. Makaretz, Michael Siao Tick Chong, Prianca Ramanan, Ana C. Amaral, Marc D. Normandin, Charles R. Vanderburg, Stephen N. Gomperts, Keith A. Johnson, Matthew P. Frosch, Teresa Gómez-Isla

**Affiliations:** 1 MassGeneral Institute for Neurodegenerative Disease, Charlestown, MA USA; 2 Department of Neurology, Massachusetts General Hospital, WACC Suite 715, 15th Parkman St., Boston, MA 02114 USA; 3 Gordon Center for Medical Imaging, Division of Nuclear Medicine and Molecular Imaging, Department of Radiology, Massachusetts General Hospital, Boston, MA USA; 4 C.S. Kubik Laboratory for Neuropathology, Massachusetts General Hospital, Boston, MA USA

**Keywords:** [F-18]-AV-1451, Flortaucipir, PET, Parkinson, Off-target binding, Basal ganglia, Choroid plexus, Microhemorrhages

## Abstract

[F-18]-AV-1451 is a novel positron emission tomography (PET) tracer with high affinity to neurofibrillary tau pathology in Alzheimer’s disease (AD). PET studies have shown increased tracer retention in patients clinically diagnosed with dementia of AD type and mild cognitive impairment in regions that are known to contain tau lesions. In vivo uptake has also consistently been observed in midbrain, basal ganglia and choroid plexus in elderly individuals regardless of their clinical diagnosis, including clinically normal whose brains are not expected to harbor tau pathology in those areas. We and others have shown that [F-18]-AV-1451 exhibits off-target binding to neuromelanin, melanin and blood products on postmortem material; and this is important for the correct interpretation of PET images. In the present study, we further investigated [F-18]-AV-1451 off-target binding in the first autopsy-confirmed Parkinson’s disease (PD) subject who underwent antemortem PET imaging. The PET scan showed elevated [F-18]-AV-1451 retention predominantly in inferior temporal cortex, basal ganglia, midbrain and choroid plexus. Neuropathologic examination confirmed the PD diagnosis. Phosphor screen and high resolution autoradiography failed to show detectable [F-18]-AV-1451 binding in multiple brain regions examined with the exception of neuromelanin-containing neurons in the substantia nigra, leptomeningeal melanocytes adjacent to ventricles and midbrain, and microhemorrhages in the occipital cortex (all reflecting off-target binding), in addition to incidental age-related neurofibrillary tangles in the entorhinal cortex. Additional legacy postmortem brain samples containing basal ganglia, choroid plexus, and parenchymal hemorrhages from 20 subjects with various neuropathologic diagnoses were also included in the autoradiography experiments to better understand what [F-18]-AV-1451 in vivo positivity in those regions means. No detectable [F-18]-AV-1451 autoradiographic binding was present in the basal ganglia of the PD case or any of the other subjects. Off-target binding in postmortem choroid plexus samples was only observed in subjects harboring leptomeningeal melanocytes within the choroidal stroma. Off-target binding to parenchymal hemorrhages was noticed in postmortem material from subjects with cerebral amyloid angiopathy. The imaging-postmortem correlation analysis in this PD case reinforces the notion that [F-18]-AV-1451 has strong affinity for neurofibrillary tau pathology but also exhibits off-target binding to neuromelanin, melanin and blood components. The robust off-target in vivo retention in basal ganglia and choroid plexus, in the absence of tau deposits, meningeal melanocytes or any other identifiable binding substrate by autoradiography in the PD case reported here, also suggests that the PET signal in those regions may be influenced, at least in part, by biological or technical factors that occur in vivo and are not captured by autoradiography.

## Introduction

[F-18]-AV-1451 (Flortaucipir) is a novel positron emission tomography (PET) tracer that preferentially binds to paired helical filament (PHF)-tau containing neurofibrillary tangles (NFTs) in Alzheimer’s disease (AD) brains [33, 51] and those that form as a function of age [31, 33]. Recent data have also shown that [F-18]-AV-1451 binding in legacy postmortem material closely correlates with NFT Braak staging and regional tau burden [34], suggesting that [F-18]-AV-1451 holds promise as a biomarker for the in vivo staging and quantification of tau pathology in AD. The affinity of this tracer for tau aggregates composed of straight filaments in non-AD tauopathy cases remains controversial [31–33, 39, 42]. Several studies, including our own, have shown that [F-18]-AV-1451 does not bind to a significant extent to β-amyloid, α-synuclein or TDP-43-containing lesions [31, 33, 42].

An increased in vivo [F-18]-AV-1451 retention in midbrain, basal ganglia and choroid plexus has been observed in a high percentage of elderly individuals regardless of their clinical diagnosis; including not only patients clinically diagnosed with AD [5, 8, 18, 40] and other non-AD tauopathies [7, 9, 11, 13, 19, 32, 38, 44, 45, 47, 49], but also patients with Parkinson’s disease (PD) and other synucleinopathies [9, 10, 21] as well as clinically normal individuals [5, 8, 9, 18, 26, 40, 45] whose brains are not anticipated to harbor tau pathology in those regions.

Our previous work using [F-18]-AV-1451 autoradiography in postmortem brain tissue revealed that the nearly universal midbrain uptake observed in vivo seems heavily influenced by the tracer off-target binding to neuromelanin-containing neurons in the substantia nigra (SN) [32, 33]. The basis for increased in vivo [F-18]-AV-1451 retention frequently seen in basal ganglia and choroid plexus, however, remains unknown. To date, only a few [F-18]-AV-1451 imaging-pathological correlation studies have been conducted on either single cases or small series of autopsy-confirmed non-AD tauopathies [27, 32, 36, 44, 46] yielding conflicting results. We have suggested that tau conformation (specifically, paired helical vs. straight tau filaments) may be critical for [F-18]-AV-1451 binding, limiting the potential usefulness of this tracer for in vivo detection of tau in many non-AD tauopathies [32, 33]. Of note, in nearly all published autopsy-confirmed non-AD tauopathy cases imaged, the highest in vivo signal and postmortem tau pathology burden were noted in basal ganglia. However, many other regions in these cases also contained high amounts of tau aggregates at postmortem but exhibited very little or no in vivo signal. These findings suggest a potential off-target binding of this tracer within brain regions of interest in many non-AD tauopathies; in particular, off-target binding in the basal ganglia would confound possible detection of tau lesions within the basal ganglia.

Literature on [F-18]-AV-1451 PET imaging in patients clinically diagnosed with α-synucleinopathies is still scarce [17, 20, 28]. A recent study reported increased in vivo tracer retention in patients with dementia with Lewy bodies (DLB) and cognitively impaired PD patients in inferior temporal cortex and precuneus that correlated well with severity of cognitive deficits [17]. Another study observed that in vivo [F-18]-AV-1451 retention is significantly lower in DLB compared to AD patients, especially in the medial temporal lobe, but elevated in posterior temporoparietal and occipital cortices relative to controls [28]. Another study showed that in vivo [F-18]-AV-1451 retention in PD patients with mild cognitive impairment is not significantly different than that of healthy controls and it does not correlate with cognitive dysfunction. Even though no imaging-pathological correlation studies have been published so far in DLB or PD patients, it is well-established the overlap of α-synuclein-containing lesions with AD pathology in many of them; something that likely accounts, at least in part, for the tracer retention observed in vivo in some of these patients [24, 25, 41].

We have had the opportunity to study in detail the [F-18]-AV-1451 imaging-pathologic correlates in an autopsy-confirmed PD case and have used this to investigate the off-target in vivo signal observed in this patient in midbrain, basal ganglia, choroid plexus and some focal areas in the cortex. Additional legacy postmortem material containing basal ganglia, choroid plexus and parenchymal hemorrhages from 20 subjects (including controls free of pathology, AD, non-AD tauopathies, DLB, vascular dementia, and cerebral amyloid angiopathy (CAA)) were also studied for comparison purposes to better understand what [F-18]-AV-1451 in vivo positivity in those regions means.

## Materials and methods

The study was approved by the local Institutional Review Board and informed consent for neuroimaging and autopsy was obtained for each subject. Demographic and postmortem data are shown in Table 1.

**Table 1:** Demographic and postmortem data from the study subjects

Case #	Postmortem diagnosis	Age at death	Gender	Braak stage for NFTs [3]	CERAD score [37]	Figure
Study case	PD	71	M	II	A	1, 2, 3
BG AD#1	AD	51	F	VI	C	4
BG AD#2	AD	90	F	VI	C	4
BG AD#3	AD	64	F	VI	C	N/A
BG AD#4	AD	86	M	VI	A	N/A
BG CTL#1	CTL	58	F	I	0	4
BG CTL#2	CTL	92	M	II	0	4
BG CTL#3	CTL	73	F	I	0	N/A
BG CTL#4	CTL	94	M	I	0	N/A
BG PSP#1	PSP	69	M	I	0	4
BG PSP#2	PSP	68	M	II	0	N/A
BG PiD	PiD	62	M	I	A	4
BG DLB	DLB	76	M	I	0	N/A
CP#1	CTE	40	M	I	0	5
CP#2	VaD	95	M	I	0	N/A
CP#3	AD	79	F	VI	C	5
CP#4	AD	73	F	VI	C	N/A
CP#5	DLB	62	M	III	B	N/A
CP#6	AD	91	F	IV	C	N/A
HE#1	CAA, AD	71	M	VI	C	6
HE#2	CAA	87	M	III	B	6

*Abbreviations*: *AD* Alzheimer’s disease, *BG* basal ganglia, *CAA* cerebral amyloid angiopathy, *CERAD* Consortium to Establish a Registry for Alzheimer’s disease, *CP* choroid plexus, *CTE* chronic traumatic encephalopathy, *CTL* control, *DLB* dementia with Lewy bodies, *F* female, *HE* hemorrhage, *M* male, *NFT* neurofibrillary tangle, *NP* neuritic plaques, *PD* Parkinson’s disease, *PiD* Pick disease, *PSP* progressive supranuclear palsy, *VaD* vascular dementia

### PET imaging

The PD subject underwent [F-18]-AV-1451 PET scan 12 months prior to death. [F-18]-AV-1451 PET (80-100 min acquisition, 4 × 5-min frames) data were acquired using a Siemens/CTI (Knoxville, TN) ECAT HR+ scanner (3D mode; 63 image planes; 15.2 cm axial field of view; 5.6 mm transaxial resolution and 2.4 mm slice interval). PET data were then reconstructed and attenuation corrected, and each frame was evaluated to verify adequate count statistics and absence of head motion. T1-weighted MRI images were acquired on a 3 T Tim Trio (Siemens) and segmented using Freesurfer (FS) as previously described [12, 14, 15]. [F-18]-AV-1451 PET images were co-registered and fused with 3 T MRI images. [F-18]-AV-1451 specific binding was expressed in FS regions of interest (ROIs) as standardized uptake value ratios (SUVR) using cerebellar grey matter as reference.

### Regional correlation between [F-18]-AV-1451 PET imaging and postmortem tissue ROIs

[F-18]-AV-1451 PET images were co-registered and fused with 3 T MRI images. A manual method was used to identify and match 31 ROIs defined on postmortem 10 mm-thick coronal brain slabs and aligned visually to the corresponding coronal T1-weighted MRI images with coregistered PET images. To optimize correspondence between ROIs defined on PET images and their pathological substrate, we used the gyral and ventricular morphology on tissue slabs to guide visual identification of matching ROIs on MRI-T1 images, using a dynamic resampling of MRI in planes of view that matched the tissue blocks planes of sectioning. ROIs were then drawn manually on MRI-T1 images using the medical software AMIDE v.1.0.5 (A Medical Image Data Examiner, http://amide.sourceforge.net) [30, 52], expanded to sample a 10 mm-slice depth at the identified ROI location and saved to represent an object map on the MRI. The object map was then used to sample the previously co-registered PET data. Mean [F-18]-AV-1451 PET-relative standardized uptake values (SUVR) in each ROI sampled were obtained.

### Brain tissue samples

In addition to the PD case, legacy postmortem brain material from 20 representative cases with various neuropathologic diagnoses (including controls free of pathology, AD, non-AD tauopathies, DLB, vascular dementia, and CAA) from the Massachusetts Alzheimer’s Disease Research Center (MADRC) Neuropathology Core were included in this study (Table 1). These additional cases were selected based on the availability of enough frozen tissue containing basal ganglia, choroid plexus, and parenchymal hemorrhages to perform autoradiography experiments. Tissue blocks from the 21 cases included in the study were fixed in formalin for 1 week before being embedded in paraffin and cut at 8-μm. The diagnostic histological evaluation in all cases included in this study was performed on 19 regions representative for a spectrum of neurodegenerative diseases in accord with published guidelines [6, 23, 35].

Frozen tissue blocks from the left hemisphere from the index case containing multiple ROIs along with blocks containing basal ganglia, choroid plexus, and parenchymatous hemorrhages from the 20 additional cases (Table 1) were sectioned in a cryostat (Thermo-Shandon SME Cryostat) into 10-μm-thick slices and used for PHF-1 immunohistochemistry (1:100, kind gift of Dr. Peter Davies), hematoxylin and Thioflavin-S staining, and [F-18]-AV-1451 autoradiography. Fresh frozen homogenates from the PD case prepared from adjacent tissue blocks were used to assess tau content in multiple ROIs by Semi-denaturing detergent agarose gel electrophoresis (SDD-AGE).

### [F-18]-AV-1451 autoradiography and quantification of tau content by semi-denaturing detergent agarose gel electrophoresis (SDD-AGE)

[F-18]-AV-1451 phosphor screen and high resolution nuclear emulsion autoradiography were performed following protocols previously described in detail elsewhere [33]. SDD-AGE was carried out according to our previously published protocol [32].

### Statistical analysis

Correlations between [F-18]-AV-1451 in vivo uptake (SUVRs) and SDD-AGE total-tau and PHF-tau measurements in different ROIs were performed using a linear regression test. Significance was set at *p* < 0.05. Statistical analysis and graphs were generated using GraphPad Prism v6.0 software (GraphPad Software Inc., La Jolla, CA).

## Results

### Clinical case description

The patient was a Caucasian male who developed progressive stiffness in his left extremities in his early 50s, along with resting tremor and bradykinesia in his left hand. He received a clinical diagnosis of idiopathic PD and had a favorable response to L-DOPA therapy. His symptoms remained stable for many years but he subsequently developed hypophonia and stuttering speech, constipation, postural instability with falls, dysphagia and occasional visual illusions. His cognition was only mildly impaired 1 year before his death (Mini-Mental State Examination score [16] of 30 and Clinical Dementia Rating [22] score of 0.5). He died at age 71.

### In vivo [F-18]-AV-1451 PET imaging

**Fig. 1:**
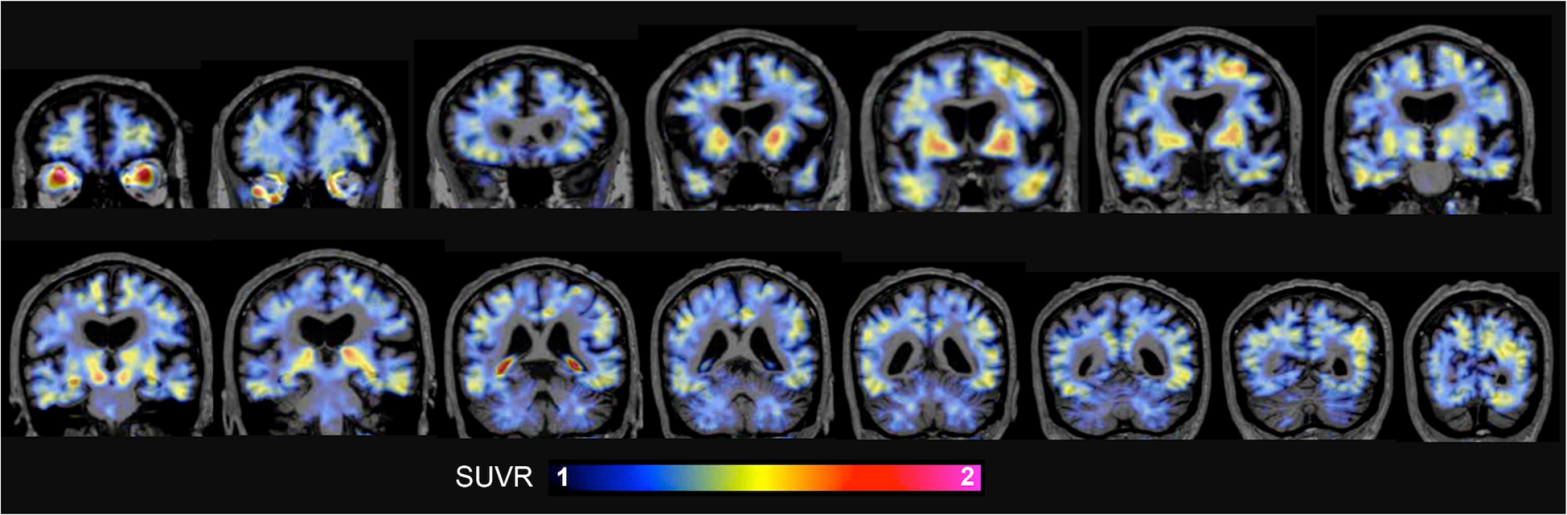
Coronal in vivo [F-18]-AV-1451 PET images of the PD subject. The color scale indicates SUVR range from 1 to 2. The subject exhibited increased tracer retention in basal ganglia, midbrain, choroid plexus and eyeballs, and milder retention in inferior temporal (bilaterally), left middle frontal and left occipital cortices. Abbreviations: PD = Parkinson’s disease; PET = positron emission tomography; SUVR = standardized uptake value ratio

A [F-18]-AV-1451 PET scan obtained 12 months prior to patient’s death showed bilateral elevated retention predominantly in midbrain, putamen and pallidum, and choroid plexus. Weaker retention was also noticed in inferior temporal (bilateral), left middle frontal and left occipital cortices (Fig. 1).

### Neuropathological examination

The autopsy revealed severe degeneration of the pigmented neurons in the SN and sparse Lewy body (LB) deposition in the SN and entorhinal cortex (EC), consistent with a diagnosis of PD Braak Stage 3 [4]. There was β-amyloid deposition in the cortex and basal ganglia consistent with Thal phase 4 [48], with predominantly diffuse plaques and sparse neocortical neuritic plaques (Consortium to Establish a Registry for Alzheimer’s disease (CERAD) score A [37]). Neurofibrillary tangles (NFTs) were confined to the EC bilaterally corresponding to Braak stage II [3]. ABC score was A3B1C1 with low likelihood of cognitive impairment due to AD according to the National Institute of Aging-Alzheimer Association scheme [23]. Mild cerebral amyloid angiopathy and moderate arteriolar sclerosis with vessel wall thickening were also present, with the latter most pronounced in the deep white matter.

### [F-18]-AV-1451 autoradiography

**Fig. 2:**
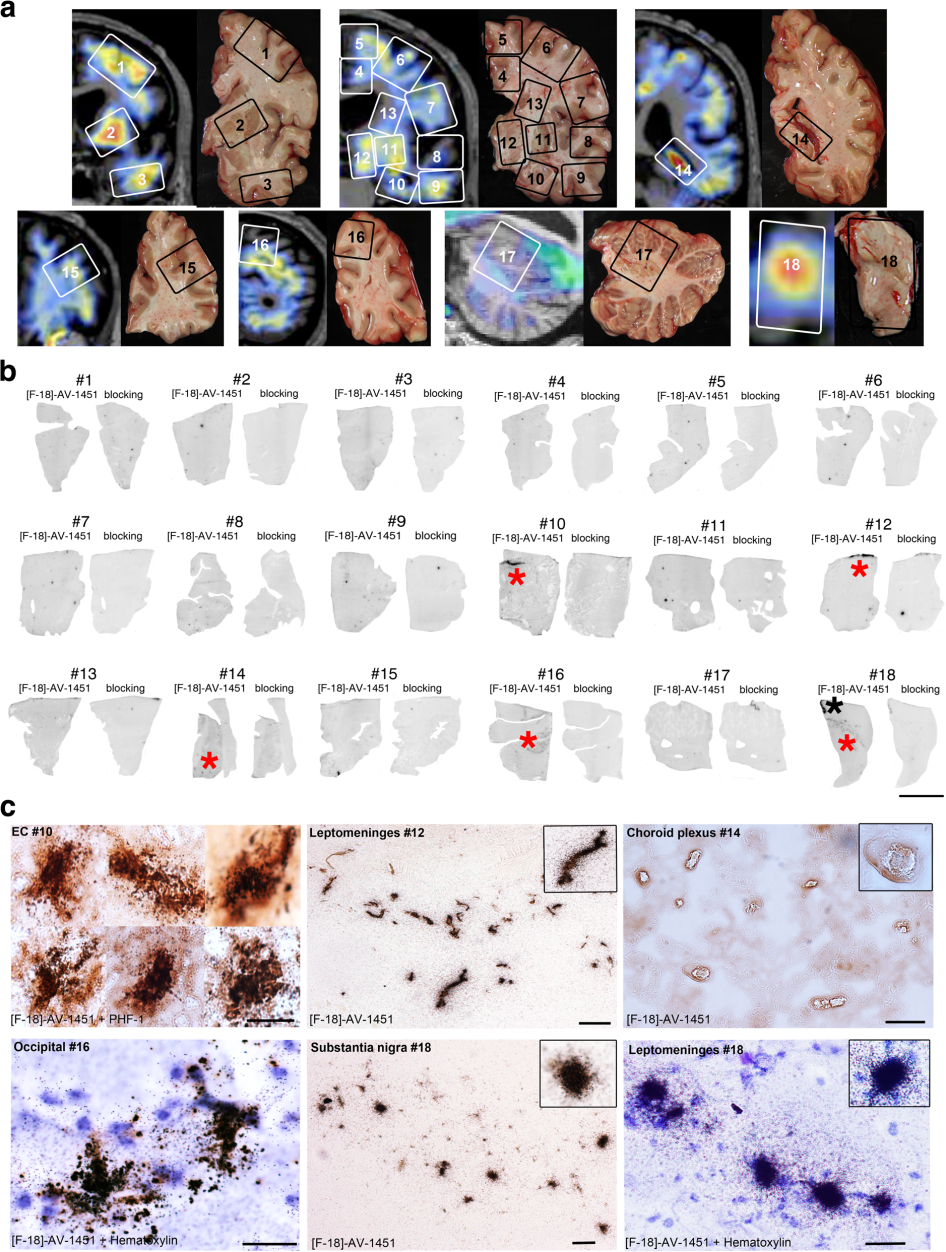
Coronal in vivo [F-18]-AV-1451 PET images superimposed to brain MRI (**a**, left), matching autopsy tissue blocks (**a**, right), phosphor screen autoradiography (**b**), and microphotographs of nuclear emulsion dipped slides after incubation with [F-18]-AV-1451 (**c**) from the PD subject. The numbers displayed on PET and autoradiography images correspond to matching ROIs. Most ROIs analyzed did not exhibit detectable autoradiography signal, with the exception of the EC (red asterisk) (#10, reflecting Braak II age-related NFT pathology), leptomeningeal melanocytes adjacent to the lateral ventricle (red asterisk) (#12), and the mesencephalus (red and black asterisks) (#18, reflecting *off-target* binding to neuromelanin-containing neurons in the substantia nigra and meningeal melanocytes, respectively). The choroid plexus (red asterisk) (#14) displayed a very faint autoradiography signal that was not blocked with 1 μM unlabeled AV-1451. Numbers correspond to the following anatomical regions: #1 = middle frontal, #2 = putamen and pallidum, #3 = inferior temporal, #4 = anterior cingulate, #5 = superior frontal, #6 = middle frontal, #7 = inferior frontal, #8 = superior temporal, #9 = inferior temporal, #10 = HPC/EC, #11 = putamen and pallidum, #12 = thalamus, #13 = caudate, #14 = choroid plexus, #15 = middle frontal, #16 = occipital, #17 = cerebellar cortex and dentate nucleus, and #18 = substantia nigra. Abbreviations: EC = entorhinal cortex, HPC = hippocampus; MRI = magnetic resonance imaging; NFT = neurofibrillary tangle; PD = Parkinson’s disease; PET = positron emission tomography; ROI = region of interest. Scale bar = 1 cm (**b**), 20 μm (**c**)

As expected, given the absence of tau pathology in this case (with the exception of incidental age-related NFTs in the EC), phosphor screen autoradiography failed to show detectable [F-18]-AV-1451 binding in most ROIs analyzed. The only exceptions, exhibiting both autoradiography signal and elevated in vivo retention, were EC (#10), substantia nigra (#18), thalamus (#12) and occipital cortex (#16) (Fig. 2a-b). High resolution nuclear emulsion autoradiography in these four regions confirmed the underlying substrate of tracer binding: NFTs in the EC (#10), neuromelanin-containing neurons in the substantia nigra (off-target, #18), leptomeningeal melanocytes (off-target, #12 and #18), and hemosiderin in a cerebral microhemorrhage (off-target, #16) (Fig. 2c).

Of note, putamen and pallidum (#2 and #11) were among the regions showing the highest in vivo tracer retention in this patient (SUVR 1.7, Fig. 2a), but exhibited no tau deposits or autoradiography signal at postmortem (Fig. 2b). Elevated in vivo retention (SUVR 1.5) was also observed in the choroid plexus (CP, #14) in the absence of tau aggregates, while this region displayed a questionable faint signal in autoradiography (not blocked with unlabeled AV-1451), and no signal in high resolution nuclear emulsion (Fig. 2a-c). A cerebral microhemorrhage was found in the section containing left occipital cortex (#16) corresponding to elevated in vivo uptake and autoradiographic binding observed in this area (Fig. 2a-c).

### Quantification of tau contents by semi-denaturing detergent agarose gel electrophoresis (SDD-AGE)

**Fig. 3:**
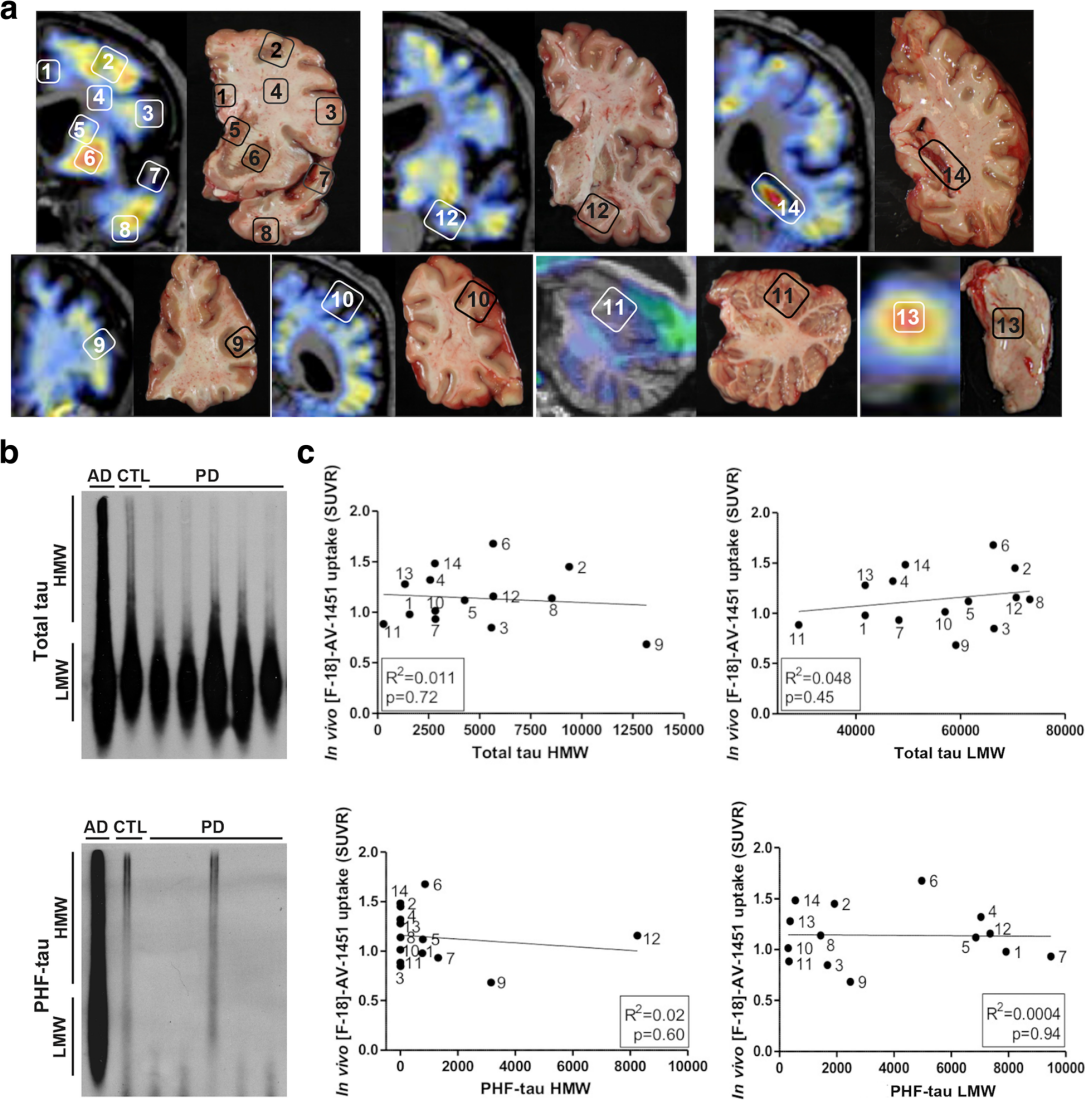
Coronal in vivo [F-18]-AV-1451 PET images (**a**, left), matching autopsy tissue blocks (**a**, right), representative images of SDD-AGE membranes stained with total tau and PHF-1 antibodies (**b**), and correlation analyses between in vivo SUVR retention values and postmortem LMW and HMW tau levels in matching ROIs (**c**) from the PD case. Numbers displayed on PET images and graphs correspond to matching ROIs. As expected, levels of total tau and phosphorylated tau, particularly in the form of HMW species, were substantially much lower in the PD case in comparison to AD brain tissue (**b**). No significant correlation was detected between in vivo [F-18]-AV-1451 signal and postmortem measurements of total tau and phospho-tau species (**c**). Numbers correspond to the following anatomical regions: #1 = anterior cingulate, #2 = medial frontal, #3 = inferior frontal, #4 = frontal white matter, #5 = caudate, #6 = putamen, # 7 = superior temporal, #8 = inferior temporal, # 9 = middle frontal, #10 = occipital, #11 = cerebellar cortex, #12 = HPC/EC and #13 = substantia nigra. Abbreviations: EC = entorhinal cortex, HPC = hippocampus, HMW = high molecular weight; LMW = low molecular weight; NFT = neurofibrillary tangles; PD = Parkinson’s disease; PET = positron emission tomography; ROI = region of interest; SDD-AGE = semi-denaturing detergent agarose gel electrophoresis; SUVR = standardized uptake value ratio

Quantification of total tau and phospho-tau by SDD-AGE in this case showed, as expected based on the neuropathologic findings, much lower levels of high molecular weight tau species when compared to those observed in cases of AD. PHF-tau levels were nearly undetectable in all ROIs examined with the exception of EC (Fig. 3b), where NFTs were present. We did not detect any significant correlation between in vivo [F-18]-AV-1451 retention and total content of tau or phospho-tau species in matched regions (Fig. 3c).

### Off-target [F-18]-AV-1451 signal in the basal ganglia

**Fig. 4:**
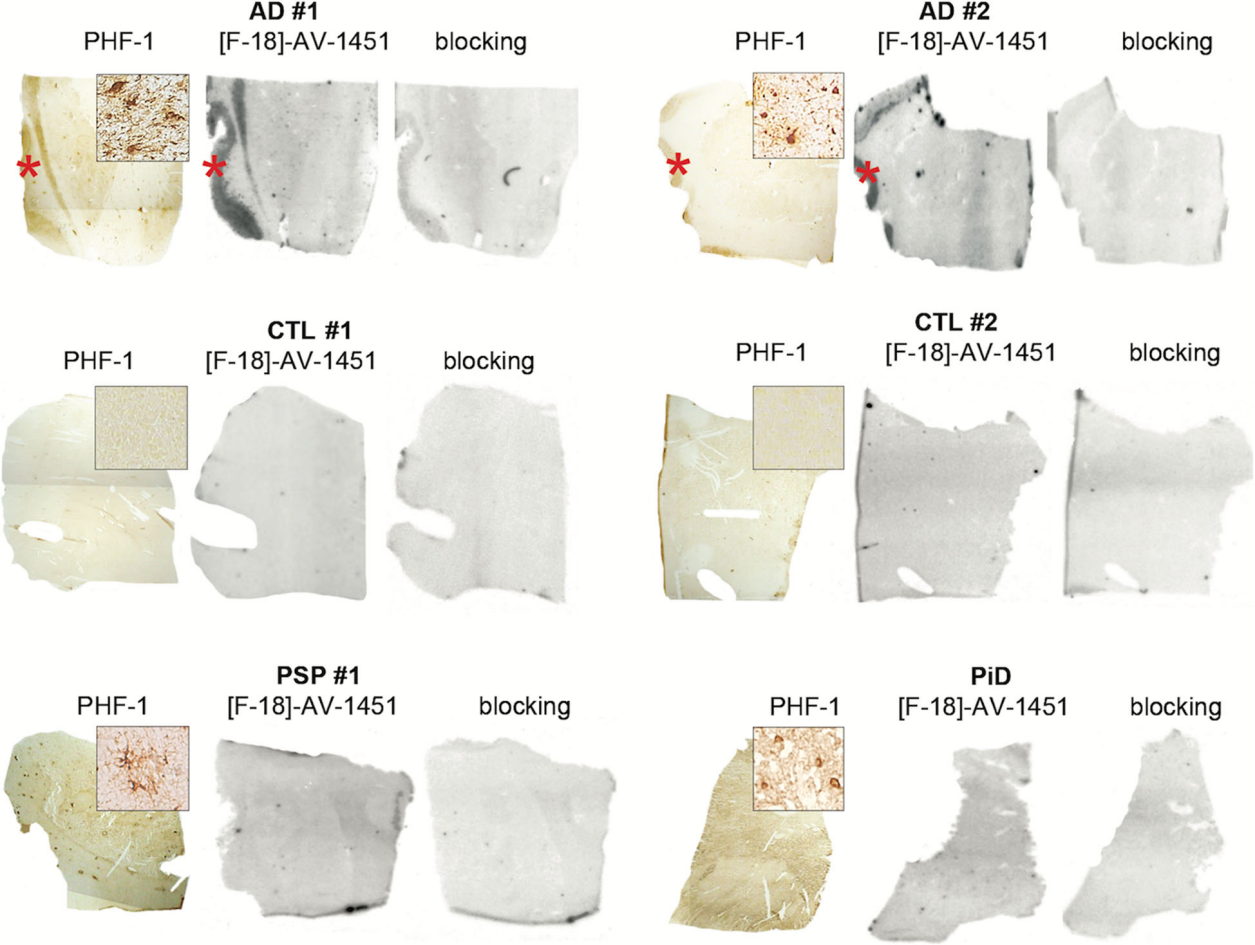
Basal ganglia tissue sections stained with PHF-1 antibody (left), [F-18]-AV-1451 autoradiography (middle) and blocking with 1 μm unlabeled AV-1451 (right) from 2 AD patients, 2 CTL and 2 non-AD tauopathy subjects. No detectable [F-18]-AV-1451 autoradiography signal was observed in the basal ganglia tissue of any of the subjects studied, including PSP and PiD subjects harboring high burden of tau deposits. AD subjects (AD#1 and AD#2) showed a strong autoradiography signal in the insular cortex adjacent to the putamen where abundant NFTs were present. Abbreviations: AD = Alzheimer’s disease; CTL = control; NFT = neurofibrillary tangles; PiD = Pick’s disease; PSP = Progressive Supranuclear Palsy

Postmortem tissue containing basal ganglia from 12 additional cases from the Massachusetts Alzheimer’s Disease Research Center (MADRC) Neuropathology Core, including 4 AD, 4 controls (CTL), 2 PSP, a Pick disease (PiD) case and a DLB case, were also examined (Table 1 and Fig. 4). Consistent with our previous observations [32, 33], no detectable [F-18]-AV-1451 autoradiography signal was observed in any of the cases including PSP and PiD cases harboring abundant tau aggregates predominantly made of straight filaments in this region. Two of the AD cases (AD #1 and #2), exhibited a positive [F-18]-AV-1451 signal in the insular cortex adjacent to the putamen reflecting the presence of abundant NFTs and thus provided an internal positive control.

### Off-target [F-18]-AV-1451 binding in the choroid plexus

**Fig. 5:**
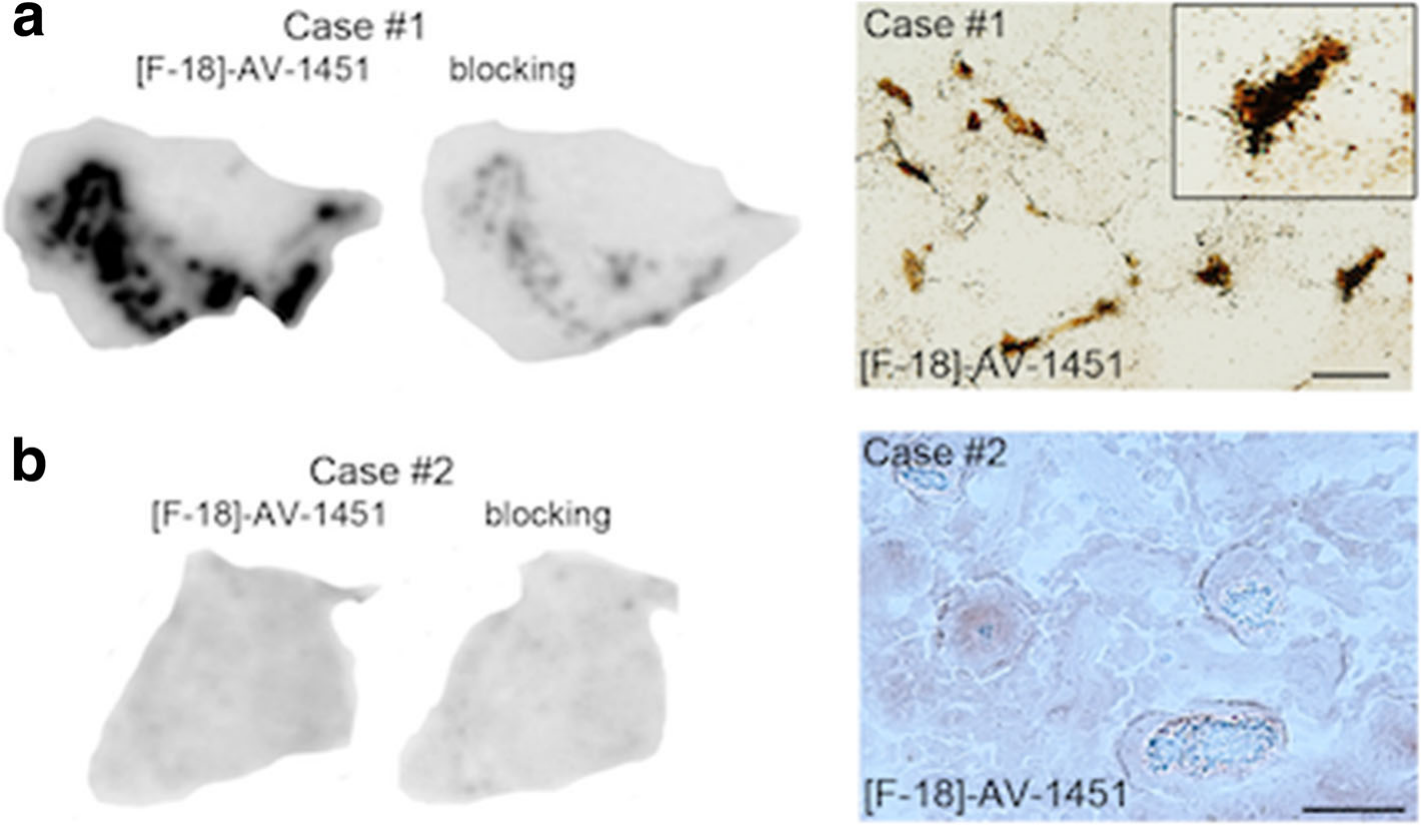
[F-18]-AV-1451 phosphor screen autoradiography (left) and nuclear emulsion autoradiography microphotographs (right) from tissue blocks containing choroid plexus of CTE (**a**, case #1) and severe brain vessel disease (**b**, case #2) subjects. Autoradiography signal was observed in case #1, corresponding to the presence of leptomeningeal melanocytes. Scale bar = 20 μm (right). Abbreviations: CTE = chronic traumatic encephalopathy

[F-18]-AV-1451 phosphor screen and high resolution nuclear emulsion autoradiography was performed on postmortem samples containing choroid plexus from 6 additional cases (Table 1 and Fig. 5). [F-18]-AV-1451 signal was noted in 3 of the cases. High resolution nuclear emulsion revealed the presence of leptomeningeal melanocytes in these 3 cases as the substrate of this signal. PHF-1 immunostaining and Thioflavin-S staining ruled out the presence of tau pathology in this region in all cases.

### Off-target [F-18]-AV-1451 binding in brain hemorrhages

**Fig. 6:**
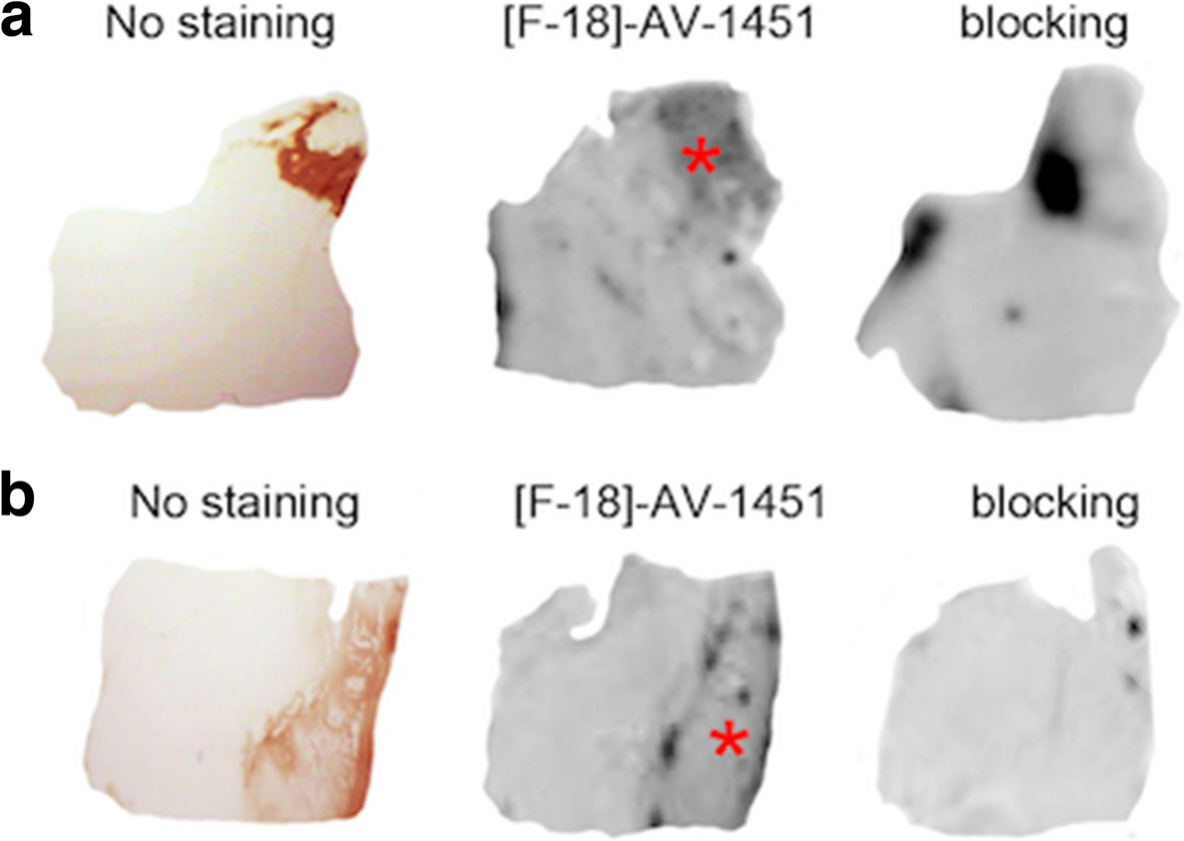
Unstained frozen tissue sections (left), [F-18]-AV-1451 phosphor screen autoradiography (middle) and blocking conditions with unlabeled AV-1451 (right) from two subjects with CAA harboring multiple brain hemorrhages (**a** and **b**). Autoradiography signal was observed in both subjects (asterisks, middle, **a** and **b**) matching the location of the hemorrhages (left, **a** and **b**). Abbreviations: CAA = cerebral amyloid angiopathy

Tissue samples from two cerebral amyloid angiopathy (CAA) cases containing multiple parenchymal hemorrhages were also included in autoradiography experiments. In agreement with our previously published observations [33], [F-18]-AV-1451 signal was noticed in both cases matching the location of the prior hemorrhages (Fig. 6).

## Discussion

This is the first imaging-pathological correlation of novel PHF-tau PET tracer [F-18]-AV-1451 in an autopsy-confirmed PD case with minimal-to-none AD co-pathology. The study of this single case has been particularly informative to learn new and valuable information about the frequently observed in vivo off-target retention of this tracer in brain regions like midbrain, basal ganglia and choroid plexus, and investigate the underlying substrate/s that may be responsible for such signal in the absence of brain tau pathology. Importantly, this same PET pattern is frequently observed in elderly individuals, including those clinically normal [5, 8, 9, 18, 26, 40, 45]. Thus, this PD case sheds light on how to correctly interpret [F-18]-AV-1451 PET in vivo images.

Our experiments using [F-18]-AV-1451 phosphor screen and high resolution autoradiography in multiple brain regions from this PD case showed that this tracer bound with strong affinity to age-related NFTs in the EC, neuromelanin-containing neurons in the substantia nigra and leptomeningeal melanocytes adjacent to the lateral ventricles, and to a lesser extent to microhemorrhages in the cortex. All these findings are consistent with our previously published observations [32, 33]. In contrast, no detectable [F-18]-AV-1451 binding was observed in basal ganglia or choroid plexus, the two regions that displayed the highest in vivo tracer retention in this case (SUVR of 1.7 and 1.5, respectively); these data are also in agreement with our prior findings [32]. The study of this PD case and additional brain material from 20 individuals with various neurodegenerative diagnoses have allowed us to further define the underlying substrates of in vivo [F-18]-AV-1451 retention in these two regions.

In our previous studies we observed robust off-target binding of [F-18]-AV-1451 to neuromelanin- and melanin-containing cells and alerted on the importance of carefully taking this finding into account when interpreting [F-18]-AV-1451 in vivo retention patterns [32, 33]. Other authors have made similar observations and suggested that this off-target binding may actually be of utility to assess dopaminergic cell loss in PD patients [21].

As noted above, elevated in vivo [F-18]-AV-1451 retention in basal ganglia has been observed in a significant proportion of elderly individuals with different clinical diagnosis, including AD [5, 8, 18, 40] and non-AD tauopathies [7, 10, 11, 13, 19, 27, 32, 36, 38, 44, 45, 47, 49], but also in cases without suspected underlying tau pathology like PD [9, 20] and MSA [10] as well as in clinically normal individuals [5, 8, 9, 18, 26, 40, 45]. Our previous studies, including correlations in 3 non-AD tauopathy cases who underwent imaging prior to death (two PSP and a MAPT P301L mutation carrier), showed elevated in vivo retention and tau pathology in basal ganglia, but no tracer binding in this region at postmortem by autoradiography, and no significant correlation between in vivo signal and tau burden in multiple ROIs [32]. The study is the largest series published to date on non-AD taupathies and made us conclude that tracer in vivo signal in basal ganglia in these cases was likely representing off-target retention in on-target areas for those diseases. The PD case studied here, with high in vivo retention but no tau-containing lesions or calcifications in this area, further reinforces this idea. Interestingly, several [F-18]-AV-1451 kinetic modeling studies [1, 2, 43, 50] have suggested that this tracer has a different kinetic profile in the putamen, with a higher initial uptake and much faster clearance in this region compared to the cortex, and enhanced retention with increasing age. It has been proposed that this may be due to additional off-target binding in the putamen or a second binding site in this region with different kinetics.

To further investigate the mismatch between elevated in vivo [F-18]-AV-1451 retention in basal ganglia and lack of autoradiography signal in this region, we performed [F-18]-AV-1451 phosphor screen and high resolution autoradiography in basal ganglia sections from 12 cases with various neurodegenerative diseases (Table 1 **,** Fig. 4). The absence of tracer binding in this region across cases, regardless of the presence or absence of tau-containing lesions suggests that the in vivo signal in this area may be due, at least in part, to non-specific biological or technical factors unrelated to tau or non-tau substrates. However, we cannot rule out with absolute certainty that the autoradiography techniques at postmortem may remove some weak [F-18]-AV-1451 labeling from the basal ganglia.

Another brain region exhibiting potential [F-18]-AV-1451 off-target retention is the choroid plexus, a highly vascular region mostly composed of an overlying specialized epithelial layer with a stroma containing blood vessels, sometimes with focal calcifications particularly in older subjects, and small rests of meningothelial elements. Elevated in vivo tracer retention was observed in the choroid plexus in the PD case reported here but, similarly to the basal ganglia, no tau pathology could be demonstrated in this area at postmortem, and autoradiography failed to show significant tracer binding. Increased in vivo retention in the choroid plexus is a common finding in a high percentage of individuals undergoing [F-18]-AV-1451 PET scans, and especially in African-Americans (Lee CM et al., communication at the Human Amyloid Imaging conference, 2017). Of note, due to the close location of choroid plexus to medial temporal lobe structures, elevated in vivo signal in this area can potentially interfere with assessment of “true” tracer retention in the hippocampus and entorhinal cortex; thus, it is important to understand the underlying substrate of tracer’s uptake in the choroid plexus. Our autoradiography study of postmortem tissue samples, which included choroid plexus from 6 individuals, detected tracer binding in three of them corresponding to the presence in these cases of abundant leptomeningeal melanocytes (see representative cases in Fig. 5a-b). These data suggest that off-target binding to melanin contributes, at least in part, to in vivo tracer retention in choroid plexus. But the PD case reported here also reveals that in vivo signal in this region may as well be present in the absence of tau pathology or melanin, pointing to an alternative substrate. It is also possible that there is a distinct kinetic profile of the compound in this area that contributes to in vivo signal but is not captured by our autoradiographic methods.

In our PD case we also noted increased in vivo [F-18]-AV-1451 retention in focal areas of frontal and occipital cortices in the left hemisphere. Our autoradiography experiments revealed, in the limited number of sections analyzed, the presence of tracer binding to an occipital microhemorrhage. It is conceivable that our PD case may harbor additional microhemorrhages that would only be revealed by extensive brain sampling. The analysis of additional legacy postmortem material from two CAA cases harboring multiple brain hemorrhages further confirmed tracer binding to those lesions in autoradiography (Fig. 6). This is in agreement with our previously published observations indicating that the off-target binding of this tracer also includes blood products [33]. Also, a recent publication describing 3 cases with probable CAA imaged with PET-[F-18]-AV-1451 showed that regions with microbleeds largely overlapped with those with increased [F-18]-AV-1451 in vivo retention [29].

## Conclusion

In conclusion, the imaging-pathologic correlation analysis of the first autopsy-confirmed PD patient who underwent [F-18]-AV-1451 PET scan prior to death confirms that this tracer not only binds with strong affinity to NFT tau pathology in AD, but also exhibits off-target binding to neuromelanin and melanin-containing cells and, to a lesser extent, to brain hemorrhagic lesions. These substrates likely explain, at least in part, the enhanced PET in vivo signal frequently noticed in midbrain, basal ganglia and choroid plexus regardless of the clinical diagnosis and of the presence or absence of tau-containing lesions in those regions. However, the robust off-target in vivo retention in basal ganglia and choroid plexus, in the absence of tau deposits, meningeal melanocytes or any other identifiable binding substrate by autoradiography in the PD case reported here, suggests that differential uptake and clearance profiles of this compound in these brain regions deserve to be further investigated. All together these data offer new important clues for the accurate interpretation of the patterns of [F-18]-AV-1451 retention observed by in vivo neuroimaging. Additional imaging-pathological studies on postmortem material from individuals studied by imaging methods prior to death will continue to provide insight into the implications of [F-18]-AV-1451 signals.
